# Targeting NLRP3 Inflammasome With Nrf2 Inducers in Central Nervous System Disorders

**DOI:** 10.3389/fimmu.2022.865772

**Published:** 2022-03-28

**Authors:** Bora Tastan, Burak I. Arioz, Sermin Genc

**Affiliations:** ^1^ Genc Laboratory, Izmir Biomedicine and Genome Center, Izmir, Turkey; ^2^ Izmir International Biomedicine and Genome Institute, Dokuz Eylul University, Izmir, Turkey; ^3^ Department of Neuroscience, Health Sciences Institute, Dokuz Eylul University, Izmir, Turkey

**Keywords:** NLRP3 inflammasome, Nrf2, inflammation, central nervous system, dimethyl fumarate, sulforaphane

## Abstract

The NLRP3 inflammasome is an intracellular multiprotein complex that plays an essential role in the innate immune system by identifying and eliminating a plethora of endogenous and exogenous threats to the host. Upon activation of the NLRP3 complex, pro-inflammatory cytokines are processed and released. Furthermore, activation of the NLRP3 inflammasome complex can induce pyroptotic cell death, thereby propagating the inflammatory response. The aberrant activity and detrimental effects of NLRP3 inflammasome activation have been associated with cardiovascular, neurodegenerative, metabolic, and inflammatory diseases. Therefore, clinical strategies targeting the inhibition of the self-propelled NLRP3 inflammasome activation are required. The transcription factor Nrf2 regulates cellular stress response, controlling the redox equilibrium, metabolic programming, and inflammation. The Nrf2 pathway participates in anti-oxidative, cytoprotective, and anti-inflammatory activities. This prominent regulator, through pharmacologic activation, could provide a therapeutic strategy for the diseases to the etiology and pathogenesis of which NLRP3 inflammasome contributes. In this review, current knowledge on NLRP3 inflammasome activation and Nrf2 pathways is presented; the relationship between NLRP3 inflammasome signaling and Nrf2 pathway, as well as the pre/clinical use of Nrf2 activators against NLRP3 inflammasome activation in disorders of the central nervous system, are thoroughly described. Cumulative evidence points out therapeutic use of Nrf2 activators against NLRP3 inflammasome activation or diseases that NLRP3 inflammasome contributes to would be advantageous to prevent inflammatory conditions; however, the side effects of these molecules should be kept in mind before applying them to clinical practice.

## Neuroinflammation and Inflammasomes

Neuroinflammation, a term used to define a wide variety of innate and adaptive immune responses within the brain and the spinal cord, contributes greatly to the pathogenesis of acute and chronic central nervous system (CNS) disorders (1). In the CNS, the primary players of innate immunity are microglia, astrocytes, and trafficking macrophages (2). These cells interact with their surrounding environment and recognize a wide range of endogenous and exogenous stimuli. These stimuli could vary from cytokines, growth factors, chemokines, adenosine triphosphate (ATP) to pathogenic signals. Among these stimuli, inflammatory signals could be grouped as Danger-Associated Molecular Patterns (DAMPs), Pathogen-Associated Molecular Patterns (PAMPs), or Homeostasis-Altering Molecular Processes (HAMPs) (3). Of note, Liston and Master recently suggested the term “HAMPs” to describe the functional consequences of pathogens on cellular processes rather than simple molecular patterns. HAMPs sense loss of cellular homeostasis of cells and initiate immune responses with or without DAMPs or PAMPs. Alteration of homeostasis within the cells, such as low potassium levels, reduced fatty acid oxidation amino acid starvation, and loss of pyrin phosphorylation, could be an inducer of HAMPs. These signals, PAMPs, DAMPs, and HAMPs, are detected by pattern recognition receptors (PRRs) which are primarily localized on innate immune cells of the CNS, especially microglia (4). The nucleotide-binding domain and leucine-rich repeat-containing receptors (NLRs) and absent in melanoma 2 (AIM2)-like receptors (ALRs), a group of cytosolic PRRs, recognize intracellular signals and cause immune response to sustain homeostasis. During this process, these receptors are involved in the formation of multiprotein complexes called inflammasomes. Once formed and activated, inflammasomes trigger proteolytic cleavage and release of pro-inflammatory cytokines, such as interleukin (IL)-1β and IL-18, and further pyroptotic cell death (5). Among all inflammasome complexes, NLR Family Pyrin Domain Containing Protein 3 (NLRP3) inflammasome is the most studied and best-characterized, as it is involved in both pathogenic and sterile inflammation activated by a wide range of signals (6).

### Structure of NLRP3 Inflammasomes

NLRP3 inflammasome is an essential component of the innate immune system, which provides defense against infections caused by bacteria, fungi, and viruses (7, 8). The NLRP3 inflammasome also recognizes DAMPs and HAMPs such as ATP, uric acid crystals, silica, asbestos, alum, and protein deposits. The NLRP3 multiprotein complex consists of three components, namely, the sensor protein NLRP3, an adaptor protein apoptosis-associated speck-like protein containing a CARD domain (ASC), and an effector pro-caspase-1 protein. There are three domains in the NLRP3 protein, that is, the NACHT domain, C-terminal leucine-rich repeats (LRRs), and the N-terminal pyrin domain (PYD) (9). It has been shown that the NACHT domain has ATPase activity which is necessary for oligomerization of NLRP3 (10). The LRR domain has both signal recognition and autoinhibition functions. The PYR domain of NLRP3 binds to the adaptor protein ASC *via* PYR-PYR interaction.

### The Mechanisms of NLRP3 Inflammasome Activation

The activation of the NLRP3 inflammasome complex is mediated by the canonical, the non-canonical, and the alternative pathways. The canonical pathway proposes a two-step activation model, the priming and the activation steps (11). The first step primes cells to induce an immune response since the intracellular levels of pro-inflammatory cytokine IL-1β and NLRP3 are insufficient to activate the inflammasome complex. The priming stage can be triggered by ligands of Toll-like receptors (TLRs) such as lipopolysaccharide (LPS), cytokines, as well as NLRs such as NOD1 and NOD2. These stimuli lead to the activation of the TLR4 receptor, which further translocates Nuclear Factor Kappa B (NF-κB) into the nucleus and initiates transcription of NLRP3 and pro-inflammatory cytokines, including pro-IL-1β (12). Recent studies suggested that in the priming step, NLRP3 protein undergoes post-translational modification such as phosphorylation and ubiquitination. JNK1-mediated S194 phosphorylation of NLRP3 is necessary for self-association and inflammasome assembly (13). In the activation step, NLRP3 recognizes various danger signals (PAMPs, DAMPs) or events causing perturbations in homeostasis, such as lysosomal disruption and mitochondrial dysfunction. These result in conformational change and oligomerization of the sensor protein NLRP3, subsequently leading to its interaction with adaptor proteins *via* its PYD domain. After that, the NLRP3-ASC complex recruits pro-caspase-1 *via* the CARD domain. The activated caspase-1 cleaves pro-IL-1β and pro-IL-18 (14). Furthermore, caspase-1 cleaves a protein called Gasdermin D (GSDMD), resulting in inflammatory programmed cell death called pyroptosis. Once cleaved, GSDMD yields two different fragments, namely C-terminal and N-terminal GSDMDs. Self-assembled N-terminal GSDMDs create pores on the plasma membrane leading to the release of the pro-inflammatory cytokines IL-1β and IL-18, loss of cellular integrity, and eventually, inflammatory cell death called pyroptosis (15).

Besides the caspase-1-mediated canonical pathway, two different pathways also mediate NLRP3 inflammasome activation. The first one, the non-canonical pathway, is mediated by catalytic activities of caspase 11 and its human homologs caspase-4 and caspase-5 (16, 17). In the non-canonical NLRP3 inflammasome activation model, TLRs are stimulated by LPS and activate NF-κB and type I interferon production, which causes the expression of caspase‐4/5/11 *via* the JAK/STAT pathway (18). Type I interferons also activate guanylate-binding proteins (GBPs) and eventually contribute to the autocatalytic activation of caspase-4/5/11 (19). Caspase-4/5/11 -mediated cleavage of GSDMD induces K^+^ efflux-dependent canonical activation of NLRP3 inflammasome complex (20). The second pathway, called alternative activation, only occurs in human and porcine monocytes. This activation pathway, unlike the others, does not depend on potassium efflux, formation of ASC speck, or pyroptotic cell death. The effector caspase-8 is induced in the TLR4–TRIF–RIPK1–FADD axis. Gaidt and colleagues demonstrated that this alternative activation pathway does not end with pyroptotic cell death (21).

### Regulation of NLRP3 Inflammasome Activation

Regulation of NLRP3 inflammasome activation can be achieved at many different levels, DNA level, transcriptional, post-transcriptional, translation, and post-translation levels. DNA methylation and histone acetylation are two mechanisms that have been shown to participate in NLRP3 regulation at the DNA level. Promoter hypomethylation of NLRP3 activates the NLRP3 inflammasome, and Histone deacetylase 6 (HDAC6) regulates NLRP3inflammasome *via* directly binding NLRP3 at the ubiquitin-binding domain (22). Aryl hydrocarbon receptor (AhR) (23), B-cell lymphoma 6 (BCL6) (24), cAMP-PKA signaling (25), AMPK-GSK3β-Nrf2 signaling (26), and Rev-erbα (27) inhibit NLRP3 inflammasome-mediated regulation at the transcriptional level. On the other hand, FADD-caspase-8 signaling pathway (28), TLR signaling pathways including TLR4/6-IRAK4/1 (29) and TLR4/Myd88/NF-κB (30), mTOR signaling pathways such as NF-κB/mTOR (31), MAPK signaling pathways like ROS/TXNIP/MAPK (32), and NF-κB/MAPK (33) activate NLRP3 inflammasome at the transcriptional level. Numerous non-coding RNA products, namely microRNAs (miRNAs) and long non-coding RNAs (lncRNAs) can regulate NLRP3 inflammasome expression at the post-transcriptional level by complementary binding to their target NLRP3 gene. Several miRNAs such as miR-7 (34), miR-22 (35), miR-30e (36), miR-133b (37), miR-223 (38) were found to block NLRP3 inflammasome. LncRNAs represent another type of non-coding RNAs that regulate the expression of their target genes. Some well-studied lncRNAs such as NEAT1 (39), Meg3 (40), HOTAIR (41), MALAT1 (42), NLRP3 (43) were shown to promote NLRP3 inflammasome activation. On the contrary, several lncRNAs like XIST (44), GAS5 (45), and SNHG7 (46) were found to inhibit NLRP3 inflammasome activation at the post-transcriptional level. Post-translational modifications are the enzymatic changes that proteins undergo to convert into a mature form. Such modifications include ubiquitination, phosphorylation, SUMOylation, alkylation, and nitrosylation. Ubiquitination of NLRP3 is accomplished by several E3 ligases and deubiquitinating enzymes including BRCC3 and BRCC36 deubiquitinate (47) and ARIH2 (48), CUL1 (49), FBXL2 (50), and TRIM31 (51) ubiquitinate NLRP3 protein. Phosphorylation by JNK1 (13), dephosphorylation by PTPN22 (52), and PP2A (53) activate the NLRP3 inflammasome. The addition of a Small Ubiquitin-like Modifier (SUMO) is another post-translational modification that helps to regulate transcription, responses to DNA damage, and hypoxic response. UBC9, a Sumo-conjugating enzyme, was shown to activate SUMO1 to SUMOylate NLRP3 and activate it while SUMO-specific protease 3 (SENP3) deSUMOylate NLRP3 for deactivation (54).

### NLRP3 Inflammasome Activation in CNS Disorders

There are many diseases of the CNS to which NLRP3 inflammasome activation is linked and involved in their etiology and pathogenesis. The activation of the NLRP3 inflammasome in both acute and chronic CNS disorders including Alzheimer’s disease (AD) (55), Parkinson’s disease (PD) (56), amyotrophic lateral sclerosis (ALS) (57), multiple sclerosis (MS) (58), neuropsychiatric diseases such as depression, schizophrenia (59), stroke, traumatic brain and spinal cord injuries (60) has been extensively studied.

AD is the most common progressive neurodegenerative disorder of the CNS, characterized by abnormal accumulation of β-amyloid plaques in the extracellular space and hyperphosphorylation of tau protein in cytoplasmic neurofibrillary tangles. Previous studies have demonstrated that both hyperphosphorylation of tau (61) and aggregation of β-amyloid peptides (62) caused activation of the NLRP3 inflammasome in microglia. Evidently, a recent postmortem study demonstrated the increased expression of ASC, caspase-1, and IL-1β in the cerebral cortex of AD patients (63). NLRP3 inflammasome is also activated in PD by α-Synuclein deposits acting as DAMPs to activate the microglial NLRP3 inflammasome. Clustered α-Synuclein leads to mitochondrial impairment, Reactive Oxygen Species (ROS) production, enhanced Cathepsin B activity, and the NLRP3 inflammasome complex formation. Furthermore, the use of genetic and MPTP-induced PD models has revealed the involvement of the microglial NLRP3 inflammasome in PD pathogenesis (34, 64–66). NLRP3 inflammasome activation by known inflammasome activators, including nigericin, aluminum potassium sulfate crystals, bacterial LPS, and vitamin K3 (menadione), leads to truncation and aggregation of α-Syn by caspase-1 in a neuronal cell model of PD (67).

Preclinical studies have demonstrated that the inhibition of NLRP3 reduces behavioral outcomes and abnormal protein accumulation associated with neurodegenerative diseases, suggesting that targeting NLRP3 inflammasome could represent a novel therapeutic approach for neurodegenerative disorders. Evaluation of the efficacy, safety, and tolerability of specific NLRP3 inflammasome inhibitors should be completed. These inhibitors could indirectly inhibit NLRP3 inflammasome activation *via* distinct mechanisms like obstruction of K^+^ efflux (Glyburide, BHB), hampering oligomerization, or binding of ASC (16673-34-0, BHB), and preventing auto-cleavage of pro-caspase-1 (FC11A-2) (68). They also can act as a direct inhibitor of inflammasome components; Caspase-1 (VX-740, VX-765, Parthenolide) or NLRP3 itself (MCC950, MNS, Oridonin, Parthenolide, OLT1177, CY-09) (68).Clinical studies on the efficacy, safety, and tolerability of these specific NLRP3 inflammatory inhibitors have started (VX-740, VX-765, MCC950, Oridonin), and the results of these studies are awaited for the application of specific NLRP3 inhibitors (69, 70). Although these developments positively impact novel therapeutic approaches, inflammasomes are part of the innate immune system and required for a proper defense system might limit their use in the future. Nevertheless, it may be possible to use naturally derived drug substances such as Nrf2 activators that inhibit NLRP3 inflammasome activation in inflammatory conditions or sterile inflammasome activation.

## Nrf2/KEAP1 Signaling Pathway

Maintaining homeostasis is indispensable to an organism’s health and survival. Environmental stressors are present everywhere and are meant to disrupt cell functions. Organisms respond and adapt to these stresses through defined regulatory mechanisms. The transcription factor nuclear factor erythroid 2-related factor 2 (Nrf2 or Nfe2l2) is a basic leucine zipper (bZIP) that belongs to the Cap ‘N’ Collar (CNC) family, and with its cytoplasmic repressor Kelch-like ECH-associated protein 1 (KEAP1), are the master regulators of redox homeostasis in the cells (71). Under non-stress conditions, Ntrf2 is localized in the cytoplasm; after activation, the nuclear translocation of Nrf2 is dependent on the balance of nuclear import and export signals. Nrf2 contains three nuclear localization sequences and two nuclear export sequences. Redox-sensitive signal leads to Nrf2 nuclear accumulation *via* blocking the interaction between nuclear exportin and nuclear export sequences. Heterodimerization of Nrf2 with Maf protein promotes nuclear retention of Nrf2. The heterodimer of Nrf2-Maf plays a master regulatory role against stress conditions by activating antioxidant-responsive element (ARE) or electrophile-responsive element (EpRE) containing genes. As one of the essential tasks of Nrf2 is to provide the necessary anti-oxidative response, it can be considered as a pioneer cell survival tool. Therefore, Nrf2 can be found dysregulated or disrupted in many diseases such as metabolic diseases, neurodegenerative diseases, aging, inflammatory diseases, cancer, and cardiovascular diseases (72).

### Nrf2/KEAP1 Structure

Nrf2 has seven Nrf2-ECH homology regions, Neh1–7, each performing different functions. Neh1 contains the CNC-bZIP region required for DNA binding and association with small musculoaponeurotic fibrosarcoma (sMaf) proteins, which are partners of Nrf2 dimerization (73). Neh2 contains highly conserved DLG and ETGE motifs involved in the interaction with the Nrf2 cytoplasmic repressor KEAP1 and seven lysine residues that serve as ubiquitylation targets (74). The C-terminal Neh3 domain, which has transcriptional activity, co-operates with Neh4 and Neh5 to upregulate Nrf2 target genes (75). Neh4 and Neh5 recruit the transcriptional co-activators CREB-binding protein (CBP) and the repressor-associated coactivator (RAC). The serine-rich Neh6 domain participates in the KEAP1-independent removal of Nrf2 through binding to the β-transducin repeat-containing protein (76). Neh7 domain suppresses Nrf2 activity by interacting with the retinoic X receptor α (77).

KEAP1 is a substrate adaptor molecule for Cul3-containing E3 ubiquitin ligase which interacts with Nrf2 and downregulates Nrf2. KEAP1 has five domains, namely N-terminal region (NTR), Broad complex Tramtrack and Bric-à-Brac (BTB) domain, intervening region (IVR), Kelch domain/double glycine repeat (DGR), and C-terminal region (CTR) (78). The DGR domain binds to DLG (latch) and the ETGE (hinge) domains of Nrf2; the IVR domain, containing specific cysteine residues, facilitates KEAP1-dependent Nrf2 ubiquitination; the BTB domain, known as homodimerization domain, also binds to Cul3-Rbx1 ligase.

### Nrf2 Activation and Regulation

Regulation of Nrf2 activation occurs at the transcriptional and post-transcriptional levels and the post-translational level *via* modification of protein stability and binding partners. KEAP1 protein is responsible for mediating the first and most important mode of regulation of Nrf2 activation. Under homeostatic conditions, two KEAP1 molecules bind to Nrf2 *via* the ETGE and DLG motifs to the Neh2 domain (79). KEAP1 functions as an adapter protein for the Cul3 E3 ubiquitin ligase, responsible for the sustained ubiquitin addition and degradation of Nrf2 (79). GSK-3 regulates Nrf2 activity by phosphorylating Nrf2 at the Neh6 domain, leading to recruitment of the ubiquitin ligase adapter β-TrCP and initiation of proteasomal degradation of Nrf2 by a Cullin1/Rbx1 complex. KEAP1-interacting region (KIR)-like ETGE motif-containing proteins such as dipeptidyl peptidase 3 (DPP3), partner and localizer of BRCA2 (PALB2), and SQSTM1/p62 contribute to Nrf2 stabilization *via* the non-canonical mechanism.

Nrf2 is activated by KEAP1-dependent and independent mechanisms. In the former mechanism, when oxidative or electrophilic stress occurs in the cell, KEAP1 is oxidized at the reactive cysteine residues, resulting in its conformational change, which in turn initiates Nrf2 dissociation from KEAP1 (79). The mechanism of Nrf2’s detachment from KEAP1 has not been fully elucidated. Two different models have been proposed to explain this mechanism, i) the hinge and latch model and ii) the quaternary model. Inhibition of GSK3-dependent phosphorylation of the Nrf2 domain, Hrd1-and WDR23-mediated ubiquitination constitute the KEAP1-independent regulation mechanisms of Nrf2 activity. The phosphorylation of Nrf2 by kinases including PKC, MAPK, PI3K, and AMPK regulates nuclear translocation and activity of Nrf2.

Nrf2 gene transcription is controlled by various transcription factors such as aryl hydrocarbon receptor (AhR) (80) and NF-κB (81). The promoter region of Nrf2 also contains an ARE-like sequence which contributes to the autoregulation of Nrf2 activity (82). Recent findings supporting that the expression of Nrf2 is epigenetically suppressed by promoter methylation still need further confirmation (83).

Nrf2 activity can be regulated at the post-transcriptional level by miRNAs and lncRNAs. In an earlier -study, miR-144, the level of which is increased in sickle cell anemia, was shown to modulate oxidative stress tolerance by targeting Nrf2 (84). Later, many miRNAs, including miR-34, miR-27a, miR-142-5p, and miR-153, were shown to participate in the regulation of Nrf2 expression by targeting Nrf2 or KEAP1 (85). As functional gene regulators, lncRNAs may also participate in the epigenetic regulation of Nrf2 activity. MALAT, ROR, ODRUL, and Nrf2-lncRNA are some of the lncRNAs shown to contribute to Nrf2 regulation in different tissues” (86). It has been shown that Nrf2 expression in human cancers can be regulated by alternative splicing. For example, alternative splicing of the *Nrf2* gene disrupts the KEAP1/Nrf2 interaction, thereby leading to enhanced Nrf2 activity (87).

## The Interplay Between NLRP3 Inflammasome and Nrf2

To develop new strategies based on Nrf2 inducers to target NLRP3 inflammasome activation in different conditions, the cross-talk between NLRP3 inflammasome and Nrf2 signaling pathways must be well acknowledged. The main link between NLRP3 and Nrf2 signaling pathways is their ability to respond to oxidative stress/ROS formation. It is known that ROS accumulation disrupts redox homeostasis and acts as an upstream signal for both NLRP3 inflammasome and Nrf2 activation due to the ROS-generated oxidative stress (88). The NLRP3 inflammasome is activated by a protein called thioredoxin-interacting protein (TXNIP), which negatively regulates the anti-oxidative thioredoxin system. ROS formation leads to binding of TXNIP to the NLRP3 and subsequent conformational change and assembly of the NLRP3 inflammasome complex (89). On the other hand, the presence of ROS or free radicals derived by ROS liberates Nrf2 from its stabilized complex with KEAP1 and causes translocation of Nrf2 to the nucleus, thereby activating transcription of detoxifying genes.

**Figure 1:**
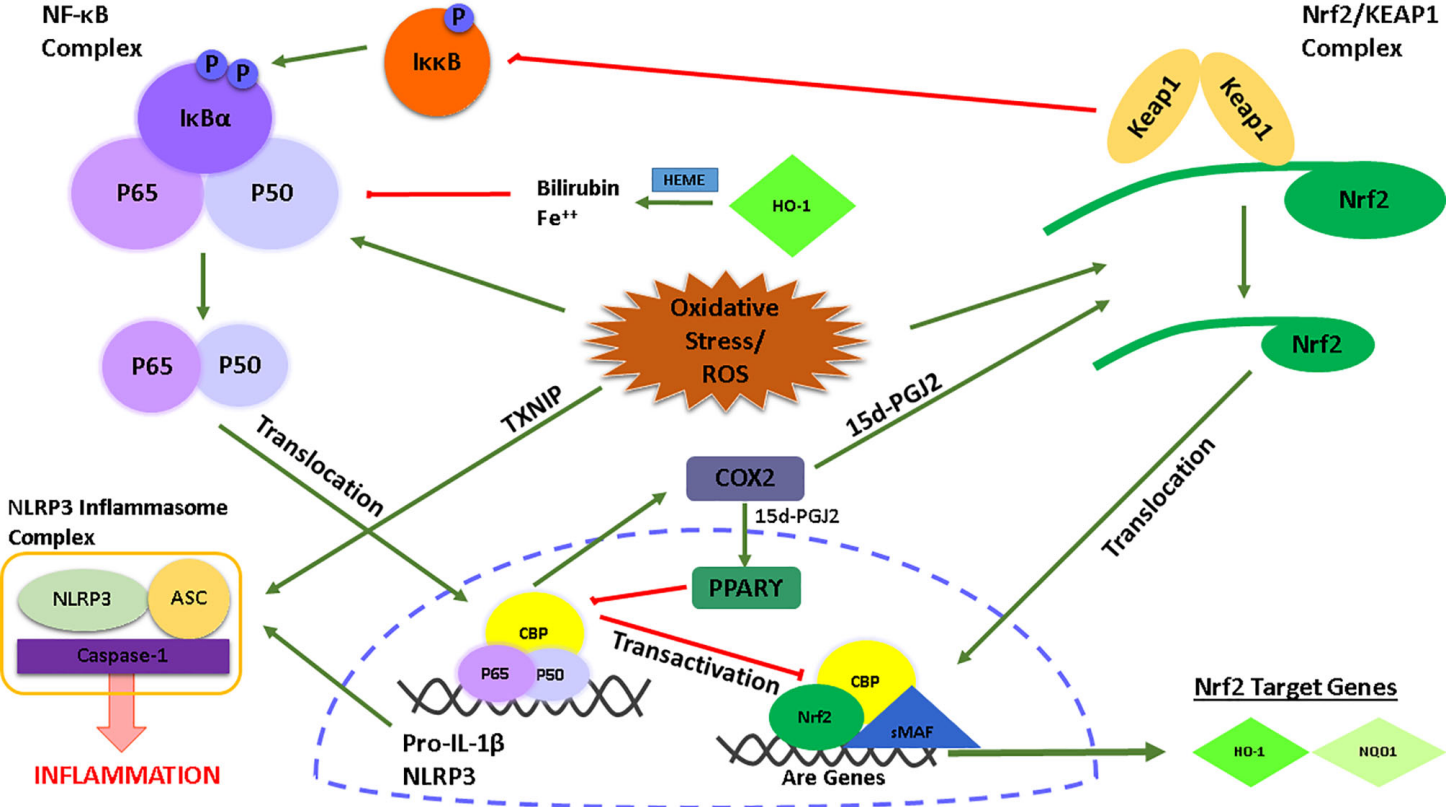
Graphical illustration of the interconnection between the NLRP3/NF-κB signaling and the Nrf2 signaling pathway.

There is also a link between Nrf2 and the transcription factor NF-κB which is an upstream signaling pathway required for the priming step of NLRP3 inflammasome activation (**Figure 1**). Several studies have reported the anti-inflammatory role of Nrf2 in NF-κB-mediated inflammation (90–92). At this point, the role of HO-1, a Nrf2 target gene, is significant. HO-1 processes heme yielding carbon monoxide, free Fe^++^, and biliverdin which is catalyzed into bilirubin (93). Free Fe^++^ and bilirubin inhibited NF-κB activity and the production of pro-inflammatory cytokines TNF-α and IL-1β. Along with HO-1, another Nrf2 target gene, NQO1, reduced NLRP3 inflammasome activation (94). Of note, in a study by Kobayashi et al., it was shown, with the usage of ChIp-Seq technology, that Nrf2 regulates the transcription of IL-6 and IL-1β by binding to the promoter regions of these NF-κB-mediated pro-inflammatory cytokines (95). Importantly, inhibition of transcription of pro-inflammatory cytokines by Nrf2 is achieved by preventing any interaction of RNA Pol II with the NF-κB complex. Along with Nrf2, KEAP1 also plays a regulatory role in NF-κB activity. After Nrf2 disassociation, KEAP1 ubiquitinates the IκB kinase β, leading to its degradation, prevention of phosphorylation of the NF-κB complex, and subsequent inhibition of NF-κB activity (96). Furthermore, the activity of NF-κB induces the release of some secondary inflammatory mediators such as COX2. A COX2 derived product, 15d-PGJ2, inhibits NF-κB *via* PPARγ (97, 98) and target cysteine residues of KEAP1 *via* its electrophilic structure (99). Lastly, there is also an inhibitory interaction between the Nrf2 and NF-κB signaling pathways. In particular, the activation of NF-κB leads to translocation of its subunits p65 and p50 to the nucleus, then p65 antagonizes with a transcriptional cofactor of Nrf2, CBP, which further prevents Nrf2 binding to and transcription of its target genes (100).

Although the protective and inhibitory effects of Nrf2 on NLRP3 inflammasome activation have been investigated (94), it has also been demonstrated the necessity of Nrf2 for appropriate activation of the NLRP3 inflammasome complex. In a study by Freigang and coworkers, it was shown that Nrf2 positively regulates NLRP3 inflammasome activation and exacerbates atherosclerosis in mice fed with a high-cholesterol diet. Furthermore, Nrf2 deficiency in the diet-induced atherosclerosis model alleviated the production of IL-1β (101). In parallel, Zhao et al. reported the essential role of Nrf2 in both NLRP3 and AIM2 inflammasome activation (102). In this study, Nrf2 was required to sufficiently activate the NLRP3 inflammasome complex and the IL-1β/caspase-1 processing. The deficiency of the Nrf2 gene also negatively affected the ASC speck formation, another indicator of the NLRP3 inflammasome activation.

## Nrf2 Inducers

Nrf2 activators could be categorized as electrophiles, protein-protein interaction (PPI) inhibitors, and multi-target drugs. Electrophiles are electrophilic molecules that alter cysteine residues of the KEAP1 protein by certain modifications like oxidation. Some electrophiles are triterpenoids like bardoxolone-methyl and RTA-408 (103), fumaric acid esters such as dimethyl fumarate (DMF) (104, 105), and monomethyl fumarate (106), organosulfurs such as oltipraz (107) and biliary acids, ursodiol (108), natural compounds including curcumin (109), resveratrol (110), quercetin (111), sulforaphane (SFN) (112), and melatonin (113). PPIs block the physical interaction of Nrf2 with KEAP1, thus activating Nrf2 and offering more selectivity compared to electrophiles like Tetrahydroisoquinoline (114), thiopyrimidine (115), naphthalene (116). One KEAP1 independent activator is GSK-3; it phosphorylates Nrf2, and thus, E3 ligase β-TrCP ubiquitinates phosphorylated Nrf2, so it is degraded. Therefore, GSK-3 inhibitors have the potential to activate Nrf2; one example is tideglusib (117). Moreover, HRD1 is an E3 ubiquitin ligase that contributes to Nrf2 degradation in a KEAP1-independent manner; LS-102 is another HRD1 inhibitor (118). There are two classes of Nrf2 inhibitors, the agonists of nuclear receptors and the natural compounds. Regarding the agonists of nuclear receptors, glucocorticoid receptor ligands like Dexamethasone (119) and clobetasol propionate (120) suppress Nrf2 transcription. Additionally, agonists of the retinoic acid receptor-α and retinoid X receptor-α, i.e., bexarotene and trans-retinoic acid, display the same effect and block Nrf2 transcription (121, 122). Several natural compounds were shown to repress Nrf2, including flavonoids luteolin (123) and wogonin (124), ascorbic acid (125), mycotoxin ochratoxin A (126), camptothecin (127), halofuginone (128), and coffee alkaloid trigonelline (129).

## Preclinical Experience With Nrf2 Inducers Against NLRP3 Inflammasome Activation in CNS Disorders

Due to their anti-oxidative and anti-inflammatory nature, Nrf2 inducers have attracted attention for inhibiting NLRP3 inflammasome activation in numerous NLRP3 inflammasome-related CNS disorders, especially the natural compounds that have been studied comprehensively. SFN, an isothiocyanate, was proved to inhibit NLRP3 inflammasome by upregulating Nrf2 in murine microglial cells (130). Isoliquiritigenin, a phenolic compound obtained from licorice, was shown to heal cognitive impairment (131) and early brain injury (132) by blocking NLRP3 inflammasome through Nr2 upregulation. In another study on the early brain injury model, mangiferin, a glucoside of norathyriol found in mango trees, was also proved to exert neuroprotective effects through Nrf2/HO-1 and NLRP3 pathways regulation (133). Furthermore, Wang et al. showed that Dl‐3‐n‐butylphthalide found in celery oil healed AD‐like pathology and suppressed NLRP3 inflammasome activation through the Nrf2‐TXNIP‐TrX signaling pathway in APP/PS1 mice (134). Celastrol, a pentacyclic triterpene from *Tripterygium wilfordi* root extract, exhibited neuroprotective properties in a MPTP-induced PD mouse model and AAV-mediated human α-synuclein overexpression in a PD model by inhibiting NLRP3 through the Nrf2 signaling pathway (135). Luteolin, a flavone, exerted cerebroprotection after subarachnoid hemorrhage (136) and neuroprotection after spinal cord ischemia-reperfusion injury (137) by preventing activation of NLRP3 inflammasome *via* the Nrf2 pathway. Astragaloside IV, a saponin found in *Astragalus membranaceus*, was found beneficial against cerebral ischemia-reperfusion injury (138), motor deficits, and dopaminergic neuron degeneration in a MPTP PD mouse model (139) *via* NLRP3/Nrf2 pathway regulation. A meroterpene from the seeds of *Psoralea corylifolia*, or bakuchiol, displayed similar effects by ameliorating cerebral ischemia-reperfusion injury *via* NLRP3/Nrf2 pathway (140). Tao et al. indicated that magnalol, biphenolic neolignane found in the bark of *Magnolia officinalis*, stimulates microglia towards the M2 phenotype to heal depressive-like behaviors through regulating Nrf2/HO-1/NLRP3 axis (141). Bixin, an apocarotenoid in the seeds of the achiote tree, was proved to diminish neuroinflammation and demyelination in an experimental autoimmune encephalomyelitis mouse model *via* upregulating Nrf2 and suppressing TXNIP/NLRP3 Inflammasome (142). Carvacrol found in the essential oil of oregano upregulates autophagy *via* the KEAP1/Nrf2/p62 axis and downregulates NLRP3, thereby providing neuroprotection in the unilateral sciatic nerve CCI model (143). Allicin, an organosulfur from garlic, prevented depressive-like behaviors in chronic social defeat stress model mouse by increasing the activities of the superoxide dismutase (SOD) and Nrf2/HO-1 pathways and blocking NLRP3 inflammasome (144). Asiatic acid is a triterpenoid from *Centella asiatica* and exhibited neuroprotective effects *via* NLRP3 inflammasome blocking and Nrf2 activation in spinal cord injury model rats (145). Dihydrolipoic acid, a reduced form of lipoic acid, remedied behavioral deficits and neuroinflammation through the Nrf2/HO-1/NLRP3 axis in a LPS rat model (146). Furthermore, saffron extract cured neuroinflammation by stimulating SIRT1, Nrf2, and HMOX1, while decreasing NLRP3 inflammasome activation in a repetitive mild traumatic brain injury model mouse (147). Neopterin, a catabolic product of guanosine triphosphate, blocks NLRP3 inflammasome activation *via* Nrf2 in human primary astrocytes (148).

Alkaloids are natural compounds produced by many plants, bacteria, fungi. Berberine, an alkaloid found in Berberis species, reduced inflammation through the TXNIP/NLRP3/Nrf2 axis in RAW 264.7 macrophages and rats (149). Another alkaloid, ephedrine, derived from plants of the Ephedra genus, was demonstrated to attenuate cerebral ischemia injury in the middle cerebral artery occlusion rat model and BV2 microglial cells *via* Akt/GSK3β/Nrf2 pathway regulation and NLRP3 suppression (150). HJ22 is a derivative of piperine, a black pepper alkaloid, heals cognitive impairment and exert cytoprotection through KEAP1/Nrf2/ARE activation and NLRP3 inhibition (151). Another piperine derivative, HJ105, recovered neuroinflammation and oxidative damage in Aβ_1-42_ AD model rats through KEAP1-Nrf2-TXNIP regulation (152). Other natural products tested against NLRP3 activation are derived from Ginseng species; for example, Ginsenoside Re, which is found in *Panax ginseng*, was shown to heal cognitive deficits in the chronic restraint stress mouse model by augmenting Nrf2 and suppressing NLRP3 (153). In addition, Pseudoginsenoside-F11 present in American ginseng restored cognitive impairment in APP/PS1 mice by regulating the Nrf2/ARE/NLRP3 pathway (154).

In addition to the abovementioned natural compounds, several hormones were demonstrated to have beneficial effects. Melatonin is a circadian rhythm regulatory enzyme with additional anti-inflammatory, antioxidant, anti-cancer functions (155). Melatonin was shown to activate Nrf2/SIRT1 against NLRP3 in murine microglial cells (113). Another study showed that melatonin enhanced brain function in chronic Gulf War Illness model rats *via* modulating the NLRP3 inflammasome through the BDNF-ERK-CREB pathway and Nrf2 alteration (156). Another hormone, adiponectin, regulated cerebral ischemia-reperfusion by blocking the NLRP3 inflammasome through regulating AMPK and GSK-3β phosphorylation and Nrf2 translocation (157). Moreover, Cheng et al. indicated that the ghrelin hormone alleviated secondary brain injury by upregulating the Nrf2/ARE pathway and blocking the NLRP3 inflammasome (158). Luo and colleagues found that N-[2-(5-hydroxy-1H-indol-3-yl) ethyl]-2-oxopiperidine-3-carboxamide, a derivative of N-acetylserotonin, prevented NLRP3 inflammasome activation through PI3K/Akt/Nrf2 signaling in a hypoxic-ischemic encephalopathy rat model (159).

Not only natural compounds but also synthetic compounds have been investigated as potential Nrf2 inducers. DMF, a synthetic fumaric acid ester, was found to prevent NLRP3 inflammasome activation through the Nrf2/NF-κB axis in N9 murine microglial cells and a LPS-induced sickness model mouse (105). Moreover, tert-butylhydroquinone (tBHQ), a synthetic phenolic antioxidant, is a well-known Nrf2 inducer; tBHQ-induced Nrf2 blocks NLRP3 inflammasome activation *via* Trx1/TXNIP in middle cerebral artery occlusion/reperfusion (MCAO/R) model rats (160). In addition, tBHQ abated NLRP3 inflammasome activation by ROS *via* Nrf2/ARE signaling in oxygen-glucose deprivation/reoxygenation (OGDR) model BV2 microglial cells (161). Moreover, multiple drugs, namely Ezetimibe (162), Ibrutinib (163), Dexmedetomidine (164, 165), Tranilast (166), Probucol (167) and various other synthetic compounds such as sodium butyrate (168), diphenyl diselenide (169), 5-(3,4-Difluorophenyl)-3-(6-methylpyridin-3-yl)-1,2,4-oxadiazole (170) and tert-butylhydroquinone (94, 160, 161), were investigated against NLRP3 inflammasome activation and Nrf2 upregulation in several *in vivo* and *in vitro* models.

## Clinical Trials With Nrf2 Inducers Against NLRP3 Inflammasome Activation in CNS Disorders

Given that inflammation, particularly NLRP3 inflammasome activation, is considered as one of the factors underlying disorders in CNS, the use of Nrf2 inducers and modulation of NLRP3 inflammasome activation could be an effective therapeutic approach against inflammatory conditions. The studies revealed that the Nrf2 signaling is dysregulated in a variety of CNS-related disorders, especially neurodegenerative diseases, such as AD & PD (171), Friedreich’s Ataxia (172), and Huntington’s disease (173). These neurodegenerative diseases fundamentally share common pathologies; ROS formation, mitochondrial dysfunction, inflammation, and disrupted homeostasis (174). Previous studies have reported different patterns of Nrf2 expression and its target antioxidant proteins in AD. Nrf2-ARE activation may occur in the early stage of disease and nuclear Nrf2 levels decrease in AD patients at a later stage (175, 176). The demonstration that Nrf2 inducer SFN provides an improvement in cognitive function and amyloid pathology in PS1V97L‐Tg mice, has supported the importance of the Nrf2/ARE pathway in Alzheimer’s disease. Postmortem studies in PD exert higher Nrf2 nuclear translocation and upregulated expression of Nrf2-regulated genes NQO1 and HO-1 in the brain of patients. Nrf2 activators DMF and MMF exerted neuroprotective effects against MPTP neurotoxicity in wild-type but not Nrf2 null mice. Demonstration of neuroprotective effects of Nrf2 activators DMF and MMF against MPTP neurotoxicity in wild-type but not Nrf2 null mice revealed the importance of Nrf2 pathway in PD pathogenesis, and stimulation of this pathway may be a therapeutic approach for PD (177).

The mechanism of action by which these inducers modulate Nrf2 may differ and primarily related to the group of Nrf2 inducers, namely electrophiles, PPI inhibitors, and multi-target drugs (178). While electrophiles interact with cysteine residues on KEAP1 by covalent bonding and cause prevention of KEAP1-orchestrated ubiquitination and subsequent degradation of Nrf2, the PPI inhibitors hinder the interaction between Nrf2-KEAP1 or KEAP1-CUL3 non-covalently (179). Lastly, multi-target drugs participate in several pathways which activate or inactivate Nrf2. In particular, GSK3 (76), which phosphorylates Nrf2 leading to its ubiquitination, BACH1 (180), which decreases the transcriptional activity of Nrf2 and subsequent expression of ARE-genes, and SQSTM1/p62 (181), which stabilize Nrf2 and assist its translocation, constitute multiple targets for Nrf2 activity. The modulation of these targets enhances Nrf2 activity and expression of ARE-genes, underscoring their possible clinical application. Numerous Nrf2 inducers with different mechanisms of action have been tested in clinical trials. Although the clinical trials have not focused on the effects of Nrf2 modulators against NLRP3 inflammasome activation parameters, the contribution of NLRP3 inflammasome activation to pathogenesis and clinical outcomes of those CNS-related disorders has been studied thoroughly (182). Among the clinically tested Nrf2 inducers, there are natural compounds such as SFN, curcumin, resveratrol, and synthetic ones such as DMF, Bardoxolone-methyl, Omaveloxolone (178). Furthermore, these compounds have been clinically tested for various disorders ranging from cancers (183) to metabolic disorders (184). The clinical trials where Nrf2 modulators were tested in different CNS-related disorders described in this section are listed in **Table 1**.

**Table 1:** The Nrf2 inducers in clinical trials.

Nrf2 Inducer	Mechanisms of Action	CNS Disorder	Clinical Progress	ClinicalTrials.gov Identifier
**Dimethyl Fumarate (DMF)**	Electrophile	Multiple Sclerosis	Approved	
		Glioblastoma	Phase I	NCT02337426
		Acute Ischemic Stroke	Phase II	NCT04891497
			Phase II	NCT04890353
			Phase II	NCT04890366
		Intracerebral Hemorrhage	Phase II	NCT04890379
**ALKS-8700**	Electrophile	Multiple Sclerosis	Phase III	NCT02634307
			Phase III	NCT03093324
**Omaveloxolone**	Electrophile	Friedreich’s Ataxia	Phase II	NCT02255435
**Sulforaphane**	Electrophile	Schizophrenia	Phase II	NCT02880462
			Phase II	NCT02810964
			Phase II	NCT01716858
			Phase II	NCT04521868
		Autism Spectrum Disorder	Phase II	NCT01474993
			Phase II	NCT02909959
			Phase II	NCT02677051
			Phase III	NCT02654743
			Phase I/II	NCT02561481
		Parkinson's Disease	Phase II	NCT05084365
		Major Depressive Disorder	Phase IV	NCT05148169
			Phase IV	NCT05145270
			Phase II	NCT04246905
		Cognitive Function	Phase II	NCT04252261
**Sulforadex**	Electrophile	Subarachnoid Hemorrhage	Phase II	NCT02614742
**Curcumin**	Electrophile	Schizophrenia/Psychosis	Phase I/II	NCT02104752
		Chronic Schizophrenia	Phase IV	NCT02298985
		Major Depression	Phase IV	NCT01750359
		Mild Cognitive Impairment	Phase II	NCT01811381
		Alzheimer’s Disease	Phase I/II	NCT00164749
			Phase II	NCT00099710
		Amyotrophic Lateral Sclerosis	Phase II	NCT04654689
		Multiple Sclerosis	Phase II	NCT01514370
**Resveratrol**	Electrophile	Friedreich Ataxia	Phase I/II	NCT01339884
			Phase II	NCT03933163
		Mild Cognitive Impairment	Phase II/III	NCT01219244
		Alzheimer’s Disease	Phase I	NCT02502253
			Phase II	NCT01504854
			Phase III	NCT00743743
			Phase III	NCT00678431
		Huntington Disease	Phase III	NCT02336633
		Depression	Phase IV	NCT03384329
		Schizophrenia	Phase II	NCT02062190
**Tideglusib**	GSK-3 inhibition	Autism Spectrum Disorders	Phase II	NCT02586935
		Alzheimer’s Disease	Phase I/II	NCT00948259
			Phase II	NCT01350362
		Amyotrophic Lateral Sclerosis	Phase II	NCT05105958
**Nordihydroguaiaretic acid**	GSK-3 inhibition	CNS Tumors	Phase I/II	NCT00404248
**Terameprocol**	GSK-3 inhibition	High-grade glioma	Phase I	NCT02575794

Among the clinically tested Nrf2 inducers, DMF, also known as BG-12 or Tecfidera^®^, is the only drug approved for Relapsing-Remitting Multiple Sclerosis (RRMS) by both Food and Drug Administration and the European Medicines Agency (185). DMF, a derivative of fumaric acid, exerts immunomodulatory, anti-inflammatory, anti-oxidative, and neuroprotective effects (186). Furthermore, Linker et al. reported that DMF acts on the cysteine 151 on KEAP1 and thus activates Nrf2 (104). a pilot study revealed that DMF could be used against RRMS (187). After that, two separate clinical trials (DEFINE and CONFIRM) demonstrated that DMF is safe for use and can effectively reduce brain lesions and relapse rates in RRMS patients (188). A recent trial which is an extension of DEFINE and CONFIRM, called ENDORSE, supported the results of previous trials in terms of efficacy and positive benefit/risk profile (189). DMF is mainly converted to MMF by intestinal esterases and distributed throughout the body (190). Therefore, an oral formulation of a MMF derivative, ALKS-8700, which exhibits improved bioavailability and efficacy, has also been tested in a phase III trial for MS (191). Due to its cytoprotective and anti-inflammatory nature, DMF is used against acute ischemic stroke and intracerebral hemorrhage, as well as glioblastoma.

Similarly, synthetic Nrf2 modulators, such as electrophilic Omaveloxolone, multi-target drugs, Tideglusib, and Terameprocol, have been developed so as to induce Nrf2 and prevent disease or decrease the clinical symptoms (178). The recently developed Omaveloxolone is another electrophilic Nrf2 inducer that modifies cysteine 151 on KEAP1 and activates Nrf2. It has been shown that Omaveloxolone, clinically tested in Friedreich’s Ataxia, can improve mitochondrial function *in vivo* (192). Furthermore, multi-target drugs, Tideglusib, Terameprocol, have also undergone clinical trials due to their modulatory function on Nrf2 *via* GSK-3 inhibition in AD (193), ALS, autism spectrum disorders (194), and CNS cancers (195).

Phytochemicals, a wide variety of chemical compounds, constitute another group of Nrf2 inducers extracted from medicinal plants and used for therapeutic purposes. Considering the innumerable phytochemicals produced by plants, these natural compounds have great potential to be used as Nrf2 modulators. The most clinically used and tested in clinical trials natural Nrf2 inducers are SFN, Curcumin, Resveratrol, and Nordihydroguaiaretic acid (178). These compounds are considered significant due to their accessibility and fair price as compared to synthetic drugs. SFN, an organosulfur compound abundantly found in cruciferous vegetables like broccoli and cabbage, is a strong electrophile modifying cysteine 151 on KEAP1. Due to its SFN’s cytoprotective features, such as anti-inflammation and anti-oxidation, it is applied in a wide range of disorders *in vitro* and *in vivo* (196). SFN is being clinically tested for various CNS-related disorders, including PD, schizophrenia, autism spectrum disorder, and depression. Furthermore, Sulforadex, an SFN-derived active compound, has been administered to patients with subarachnoid hemorrhage (197). Likewise, electrophilic polyphenolic compounds, curcumin extracted from turmeric (*Curcuma longa*), and resveratrol derived from nuts and berries are the most clinically tested compounds after SFN. Because of their anti-oxidative and anti-inflammatory properties, as well as their ability to react with KEAP1 *via* its cysteine 151 residue, they could serve as potent Nrf2 modulators (11, 185). They have been clinically tested in commonly occurring neurodegenerative diseases such as AD, Mild Cognitive Impairment, ALS, Huntington’s disease, neuropsychiatric disorders such as schizophrenia, depression, MS, and Friedreich’s Ataxia.

Apart from being tested clinically, as mentioned before, DMF is the only Nrf2 inducer approved exclusively for CNS-related disorders. Although the safety and efficacy of DMF have been shown with large cohorts and even in pregnant women (198), it causes substantial side effects such as a reduced number of lymphocytes (199). DMF or other electrophilic Nrf2 activators induce off-target side effects, which has been explained by S-alkylation of cysteine thiols non-specifically and subsequent deficiency of anti-oxidative glutathione (200). These concerns are valid for both synthetic and natural compounds as the active compounds, electrophilic or other groups of Nrf2 inducers, especially when used at high doses, activate not only the Nrf2 signaling cascade but a considerable number of distinct signaling pathways. However, there are ongoing studies, which require great effort, time, and expenses, focusing on the modulation of Nrf2 as a potential therapeutic approach against diseases with improved efficiency and less off-target effects.

## Concluding Remark

Since the discovery and molecular/biochemical elucidation of the NLRP3 inflammasome by Tschopp and his colleagues in 2002, cumulative evidence acquired by preclinical (*in vitro* and *in vivo*) and clinical studies indicates that the contribution of NLRP3 inflammasome activation to a wide variety of diseases remains largely unelucidated. Furthermore, the ablation of NLRP3 inflammasome-related genes or small inhibitors against the NLRP3 inflammasome complex alleviates the progress, severity, or clinical outcomes of NLRP3-associated diseases. Therefore, the need for developing therapeutic strategies targeting the structural integrity or activity of the NLRP3 inflammasome complex is indispensable. Although developing novel drugs or repurposing currently used drugs might be effective in terms of specificity, the pharmacological modulation of cytoprotective signaling pathways might be another therapeutic approach to suppress the NLRP3 inflammasome activation and subsequent cell death. Since NLRP3 inflammasome senses ROS, inflammatory cytokines, and endogenous metabolites, which damage homeostasis as a regulator of redox, metabolism, and inflammation, Nrf2 represents a powerful candidate for targeting the NLRP3 inflammasome signaling cascade.

Given that Nrf2 regulates more than 250 homeostatic genes, Nrf2 plays a pivotal role in cytoprotection. This role is also supported by the presence of a plethora of naturally derived and designed Nrf2 inducers, which modulate Nrf2 and enhance its cytoprotective effects. Furthermore, some Nrf2 inducers, such as DMF and SFN, have undergone trials in preclinical and clinical models of diseases with which NLRP3 inflammasome activation is associated. As the recent advances and studies enhance our knowledge on the NLRP3/Nrf2 crosstalk, the need to establish specific therapeutic approaches with precision using Nrf2 inducers increases. To achieve this, more comprehensive and multidisciplinary research efforts are needed. Nevertheless, this research will be translated and broaden our current insight into the regulation of NLRP3 inflammasome and its related diseases.

## Author Contributions

BT and BA wrote the first draft of the manuscript. SG revised and approved the submitted version of the manuscript. All authors contributed to the article and approved the submitted version.

## Conflict of Interest

The authors declare that the research was conducted in the absence of any commercial or financial relationships that could be construed as a potential conflict of interest.

## Publisher’s Note

All claims expressed in this article are solely those of the authors and do not necessarily represent those of their affiliated organizations, or those of the publisher, the editors and the reviewers. Any product that may be evaluated in this article, or claim that may be made by its manufacturer, is not guaranteed or endorsed by the publisher.

## Abbreviations

AD, Alzheimer’s Disease; ALS, Amyotrophic Lateral Sclerosis; ARE, Antioxidant Response Element; ATP, Adenosine Triphosphate; ASC, Apoptosis-associated Speck-like Protein containing a CARD; CBP, CREB-binding protein; CNS, Central Nervous System; DAMPs, Danger-Associated Molecular Patterns; DMF, Dimethyl Fumarate; GSDMD, Gasdermin D; HAMPs, Homeostasis-Altering Molecular Processes; IL, Interleukin; KEAP1, Kelch-like ECH associated protein; LncRNA, Long Non-coding RNA; LPS, Lipopolysaccharide; miRNA, MicroRNA; MS, Multiple Sclerosis; NF-κB, Nuclear Factor Kappa B; NLRP3, NLR Family Pyrin Domain Containing Protein 3; Nrf2, Nuclear Factor Erythroid 2-related Factor 2; PAMPs, Pathogen-associated molecular patterns; PD, Parkinson’s Disease; PPI, Protein-Protein Inhibitor; PRR, Pattern Recognition Receptors; ROS, Reactive Oxygen Species; RRMS, Relapsing-Remitting Multiple Sclerosis; SFN, Sulforaphane; TLR, Toll-like Receptor; TXNIP, Thioredoxin-interacting protein.
